# Circadian Regulation of the Plant Transcriptome Under Natural Conditions

**DOI:** 10.3389/fgene.2019.01239

**Published:** 2019-11-29

**Authors:** Paige E. Panter, Tomoaki Muranaka, David Cuitun-Coronado, Calum A. Graham, Aline Yochikawa, Hiroshi Kudoh, Antony N. Dodd

**Affiliations:** ^1^Department of Cell and Developmental Biology, John Innes Centre, Norwich, United Kingdom; ^2^Center for Ecological Research, Kyoto University, Otsu, Japan; ^3^School of Biological Sciences, University of Bristol, Bristol, United Kingdom

**Keywords:** circadian rhythms, plant sciences, transcriptomics, environmental signaling, signal integration, phenology, *Arabidopsis*

## Abstract

Circadian rhythms produce a biological measure of the time of day. In plants, circadian regulation forms an essential adaptation to the fluctuating environment. Most of our knowledge of the molecular aspects of circadian regulation in plants is derived from laboratory experiments that are performed under controlled conditions. However, it is emerging that the circadian clock has complex roles in the coordination of the transcriptome under natural conditions, in both naturally occurring populations of plants and in crop species. In this review, we consider recent insights into circadian regulation under natural conditions. We examine how circadian regulation is integrated with the acute responses of plants to the daily and seasonally fluctuating environment that also presents environmental stresses, in order to coordinate the transcriptome and dynamically adapt plants to their continuously changing environment.

## Introduction

The Earth rotates on its axis approximately every 24 h. This causes daily cycles in a variety of environmental conditions, such as the presence, spectrum and direction of light, ambient temperature, relative humidity, and the activity of herbivores. Furthermore, the axial tilt of the Earth causes seasonal changes in the photoperiod and other environmental parameters. The daily environmental cycles appear to have driven the evolution of circadian rhythms. Circadian rhythms are biological cycles that have a period of about 24 h and persist in the absence of external cues. A key feature of circadian rhythms is that they define the daily phase of biological processes, and therefore organize the daily timing of the transcriptome so that cellular processes occur at appropriate times of day and in a coordinated manner (Harmer et al., 2000). Circadian regulation increases the performance and fitness of higher plants, including crops (Green et al., 2002; Dodd et al., 2005; Turner et al., 2005; Graf et al., 2010; Izawa et al., 2011; Müller et al., 2015).

The molecular functioning of plant circadian rhythms and the circadian organization of the transcriptome has been investigated predominantly under laboratory conditions. However, circadian rhythms are thought to have evolved as an adaptation to naturally fluctuating environments, and an understanding of their importance for ecosystems and field-grown crops requires knowledge of circadian regulation under natural conditions. Here, we explore the recent expansion of understanding of molecular and cellular aspects of circadian regulation under natural conditions, with a focus on the regulation of the transcriptome.

### Features of Circadian Rhythms

The circadian system in plants is often considered to include several conceptual components. First, input or entrainment pathways adjust the phase of the circadian oscillator to match the phase of the environment by changing the circadian phase in response to specific environmental cues known as zeitgebers. Second, the circadian oscillator is a molecular network that produces an estimate of the time of day that, in plants, consists of an interconnected network of genes and proteins arranged in feedback loops. Third, output mechanisms communicate the measure of the time of day generated by the circadian oscillator to clock-controlled processes in the cell. Finally, signaling pathways that entrain the circadian clock and others that participate in environmental responses are, themselves, circadian regulated so that the magnitude of their response to a defined stimulus depends upon the time of day. This aspect of circadian regulation is known as the circadian “gating” of signal transduction (Hotta et al., 2007). Next, we summarize key features of circadian regulation in *Arabidopsis thaliana*, and the reader is referred to other review articles for greater depth (Nagel and Kay, 2012; Hsu and Harmer, 2014; Huang and Nusinow, 2016; Millar, 2016). There are differences in circadian clock architecture between *Arabidopsis* and crop species, which are summarized in a number of excellent articles (Song et al., 2010; Bendix et al., 2015; Nakamichi, 2015). Key differences between *Arabidopsis* and crops for consideration include effects of polyploidy, genome duplication and hybrid vigor (Ermolaeva et al., 2003; Ni et al., 2009; Hotta et al., 2013; Liu et al., 2014), allelic diversity (Xie et al., 2015), and species- and variety-specific alterations in the relationship between the circadian clock and control of flowering time (Turner et al., 2005; Cockram et al., 2007; Song et al., 2015).

### Mechanisms of Circadian Regulation in Plants

Entrainment is the process whereby the phase of the circadian oscillator is adjusted to match the daily phase of the environment. This is crucial to allow the oscillator to provide a faithful biological estimate of the time of day. In *Arabidopsis*, entrainment occurs in response to light, temperature, and metabolic cues (Somers et al., 1998; Salomé and McClung, 2005; Kim et al., 2007; Haydon et al., 2013). The entrainment mechanism involves the phytochrome, cryptochrome, and other LOV-domain photoreceptors such as ZEITLUPE (ZTL) (Somers et al., 1998; Kim et al., 2007), temperature responses mediated by PRR7, PRR9, and ELF3 (Salomé and McClung, 2005; Thines and Harmon, 2010), and metabolite signaling by sugar-sensing protein kinases such as SNF1-RELATED PROTEIN KINASE1.1 (KIN10/AKIN10/SnRK1.1) and also PHYTOCHROME INTERACTING FACTOR (PIF) proteins (Haydon et al., 2013; Shin et al., 2017; Shor et al., 2017; Frank et al., 2018). Entrainment in *Arabidopsis* generally takes the form of parametric entrainment, whereby the pace of the oscillator is transiently accelerated or decelerated in order to adjust the circadian phase (Covington et al., 2001; Michael et al., 2003; Rémi et al., 2010). This contrasts nonparametric entrainment, which involves an instantaneous reset of the circadian phase. Effects upon the circadian oscillator of factors such as relative humidity (Mwimba et al., 2018), phytohormones (Hanano et al., 2006) and ROS (Lai et al., 2012) might allow these factors to entrain the circadian oscillator, although whether they are genuinely zeitgebers would benefit from further assessment by formal approaches such as the construction of phase response curves.

The circadian oscillator is formed from transcription/translation loops that are connected primarily by repressive feedback (Hsu and Harmer, 2014), augmented by post-translational and epigenetic mechanisms. Over 20 circadian clock-related genes have been identified in *Arabidopsis*, with homologs of these present in other plants including crops. CIRCADIAN CLOCK-ASSOCIATED1 (CCA1) and LATE ELONGATED HYPOCOTYL (LHY) are MYB-like transcription factors forming part of a core feedback loop within the circadian oscillator (Schaffer et al., 1998; Wang and Tobin, 1998). *CCA1* and *LHY* transcript abundance peaks in the morning and decreases throughout the day. CCA1 and LHY repress the expression of the evening-phased *TIMING OF CAB EXPRESSION1* (*TOC1*) by binding to its promoter (Harmer et al., 2000; Lu et al., 2009; Yakir et al., 2009). Inversely, TOC1 regulates *CCA1* and *LHY* (Alabadí et al., 2001) by binding to their promoters to repress their expression (Gendron et al., 2012; Huang et al., 2012). CCA1 and LHY also repress other evening-phased genes, including *GIGANTEA* (*GI*), *LUXa ARRHYTHMO* (*LUX*), *BROTHER OF LUX ARRHYTHMO* (*BOA*), *EARLY FLOWERING3* (*ELF3*), and *ELF4* (Dai et al., 2011; Lau et al., 2011; Lu et al., 2012; Nagel and Kay, 2012). Furthermore, CCA1 and LHY appear to repress day-phased *PRR9* and *PRR7* in a time of day-dependent manner (Kamioka et al., 2016; Adams et al., 2018). *PRR9* reaches peak abundance after dawn, followed by *PRR7*, and these act as *CCA1* and *LHY* repressors along with their homolog PRR5. Furthermore, PRR-family proteins repress transcription of the morning-phased *REVEILLE8* (*RVE8*), which is within the same protein family as CCA1 and LHY (Rawat et al., 2009; Nakamichi et al., 2010; Farinas and Mas, 2011; Nakamichi et al., 2012; Farré and Liu, 2013). In contrast to the morning *RVE8* transcript peak, RVE8 protein levels peak during the afternoon and RVE8 appears to positively regulate hundreds of evening-phased genes by binding to evening element (EE) promoter motifs to induce their transcription (Rawat et al., 2011; Hsu et al., 2013). *RVE4* and *RVE6* are close homologs to *RVE8* that might function redundantly with RVE8 (Hsu et al., 2013). A further component of the circadian oscillator is the evening complex (EC), incorporating LUX, ELF3, and ELF4. Within this, ELF3 and ELF4 negatively regulate the morning gene *PRR9* and induce *CCA1* expression by interacting with BOA (Dai et al., 2011; Dixon et al., 2011; Helfer et al., 2011; Nusinow et al., 2011; Herrero et al., 2012).

Circadian-regulated *cis* elements within gene promoters appear to couple the circadian oscillator with the rhythmic regulation of the transcriptome. Therefore, in addition to forming parts of the circadian oscillator, circadian oscillator components are positioned within output pathways to provide transcriptional regulation of circadian-regulated genes. There is a rich set of circadian-regulated *cis* elements within the promoters of the *Arabidopsis* genome, and these confer distinct circadian phases to distinct subsets of transcripts (Harmer et al., 2000; Covington and Harmer, 2007). For example, the circadian oscillator components CCA1 and LHY are thought to bind to the promoters of at least 439 (CCA1) and 722 genes (LHY), respectively (Nagel et al., 2015; Kamioka et al., 2016; Adams et al., 2018). Similarly, interaction between circadian oscillator components and other signaling proteins, such as the PIF proteins involved in environmental signaling, can change their promoter-binding activity and allow the integration of circadian and environmental cues (Martín et al., 2018).

### Environmental Differences Between Laboratory and Field Conditions Will Impact Circadian Regulation

**Figure 1:**
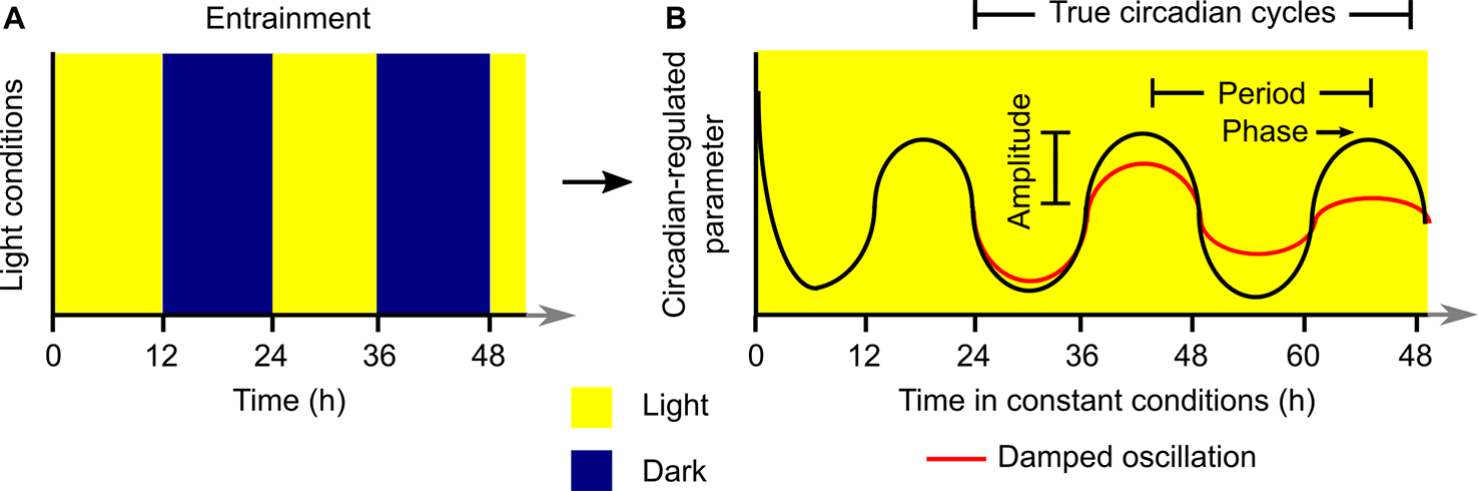
Generalized structure for execution of an experiment to investigate properties of a circadian rhythm under laboratory conditions. The plant is **(A)** entrained to a square-wave light/dark (or warm/cold) cycle and subsequently **(B)** transferred to constant conditions where the circadian-regulated feature of the plant will free-run. Analysis of the resultant rhythm **(B)** with a variety of quantitative tools can be used to extract information about the circadian period, phase, and amplitude. Data collected during the first 24 h of constant conditions is not considered to be a true circadian cycle, and these data are often discarded prior to quantitative analysis of free-running rhythms.

Laboratory investigations of plant circadian rhythms commonly involve the entrainment of plants to square-wave light/dark cycles in the presence of uniform temperature conditions (**Figure 1A**). The plants are transferred subsequently to constant light and temperature conditions for investigation of the rhythmic process under question (**Figure 1B**). Under permissive constant conditions, the circadian-regulated process will “free run” and its characteristics (e.g., circadian period, phase, damping) can be evaluated and quantified (**Figure 1B**). Plants can be exposed to a variety of other types of entrainment conditions, such as temperature fluctuations, which are usually also imposed with square-wave patterns. For this analysis, it is common to discard data collected during the first 24 h of constant conditions because this often includes transient responses to the final dawn that are not representative of the subsequent free-running rhythm (**Figure 1B**) (Dodd et al., 2014).

**Figure 2:**
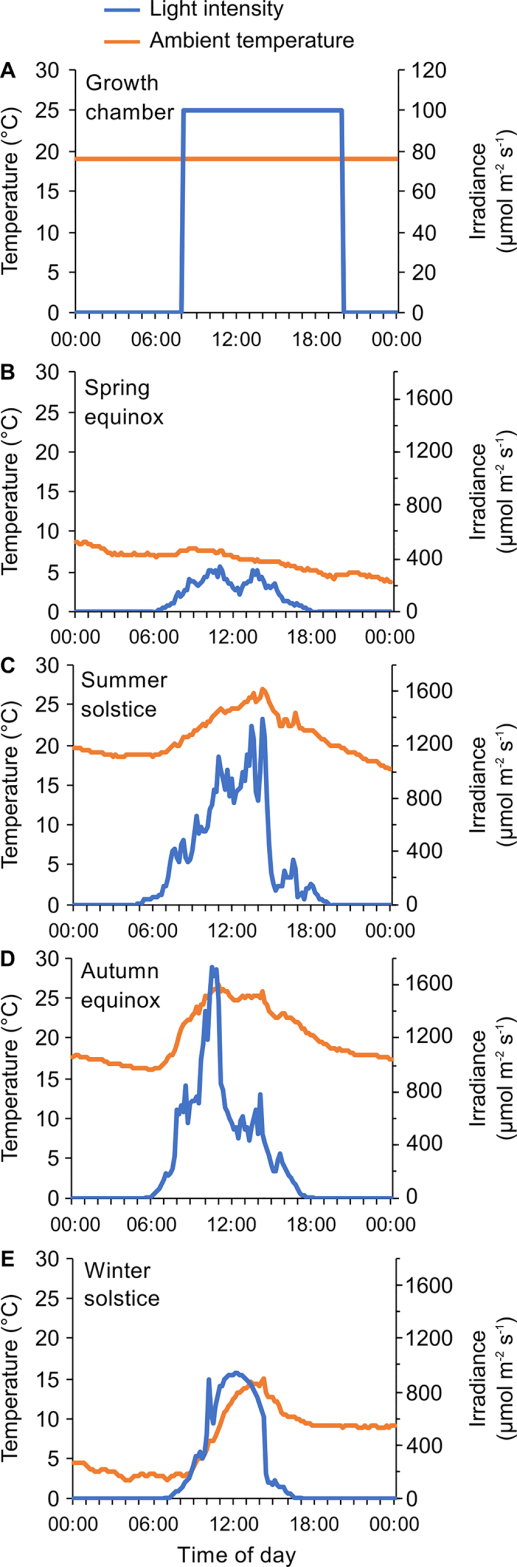
Examples of differences in environmental parameters that will impact circadian rhythms of plants in growth chamber and field conditions. **(A)** Typical entrainment program used in the laboratory to study rhythmic processes, characterized by a 12-h light/12-h dark square-wave cycle of illumination combined with constant temperature conditions. **(B–E)** Light and temperature conditions in a naturally-fluctuating environment, monitored for 24 h during four cardinal points of the year. In this case, the environment is characterized by gradual daily changes in light and temperature conditions, seasonal differences in the temperature and light environment, and short-term variations caused by altering weather conditions. Data were collected during 2018–2019 at a field site equipped with a meteorological station, Taka-cho, Nishiwaki, Hyogo Prefecture, Japan (35° 05’ N, 134° 54’ E) (Kudoh et al., 2018; Nagano et al., 2019).

These structured environmental conditions are important for investigating the architecture and function of the circadian oscillator, and the downstream circadian regulation of transcription, metabolism, development, and physiology. However, plants growing under natural conditions are unlikely to experience conditions of continuous light, darkness, constant temperature or square-wave light/dark cycles (**Figures 2A–E**). There are also differences in environmental conditions caused by the weather, which are superimposed on the 24-h cycle. For example, the temperature can differ between successive days (e.g., **Figures 2B, E**). Therefore, understanding the contribution of the circadian oscillator to temporal structures in plants growing under natural conditions requires knowledge of the functioning of the circadian oscillator under either controlled conditions that fluctuate in a manner more representative of natural environments, or under naturally occurring field conditions (**Figures 2B–E**). Key differences between commonly used laboratory conditions (**Figure 2A**) and those experienced by plants in nature include gradual changes in irradiance at dawn and dusk, unpredictable changes in light and temperature conditions due to the weather (**Figures 2A, B**). There are also progressive seasonal changes in photoperiod, irradiance, and temperature (**Figures 2B–E**). While there have been comparisons of differences between plants grown in growth chambers and field conditions (Limpens et al., 2012; Poorter et al., 2016), these studies have not considered the impacts upon circadian regulation of these differences. Because a number of environmental parameters that differ between chamber and field conditions are key entrainment cues for the clock, there are likely to be fascinating differences in circadian function under field conditions that add depth to our understanding of plant circadian regulation. Here, we focus on the interactions between circadian and abiotic environmental cues that regulate the transcriptome under natural conditions.

### Effects of Light Conditions in Natural Environments Upon Plant Circadian Regulation

Plants in natural environments experience fluctuating light levels during the course of the day due to changes in cloud cover and shading by neighbouring plants (**Figures 2B–E**). These fluctuations affect the intensity as well as the wavelengths of light that are incident upon plants. This is likely to alter entrainment, because increases in light intensity shorten the circadian period in Arabidopsis (Somers et al., 1998). Both red and blue light provide entraining inputs to the circadian clock (Millar et al., 1995; Somers et al., 1998). In *Arabidopsis,* red light is sensed by the phytochromes phyA-E, while blue light is sensed by the cryptochromes cry1 and cry2. However, because plants are normally exposed to white light, combined information from these and other photoreceptors will influence the circadian oscillator (Oakenfull and Davis, 2017). In the laboratory, continuous far red light causes a shorter circadian period, so it is possible that under natural conditions the ratio of red to far red light (R:FR), which is altered by shade, the threat of shade, and the angle of incident light, will influence circadian regulation (Wenden et al., 2011). However, there is little information concerning the effect of R:FR more representative of natural environments upon the circadian oscillator. In addition to the phototransduction pathways that act upon the circadian oscillator through the photoreceptors, photosynthetic light harvesting leads to the production of photosynthetic sugars and reactive oxygen species (ROS). Photosynthetic sugars can entrain the circadian oscillator (Haydon et al., 2013; Frank et al., 2018) and there is evidence that ROS can adjust circadian oscillator function (Lai et al., 2012), so a fascinating area for future investigation would be to identify the relevance of these mechanisms that couple metabolism with circadian regulation under natural conditions.

Under square-wave environmental fluctuations, the transition from dark to light and *vice versa* is instantaneous, while under natural conditions plants experience a gradual increase and decrease in light intensity at dawn and dusk, respectively (**Figure 1**). A comparison of plant growth under square-wave and naturally fluctuating light environments identified that plants grown under fluctuating light have thinner leaves, decreased light absorption by leaves, and a decrease in carbon assimilation compared with plants grown under square-wave light/dark cycles (Vialet-Chabrand et al., 2017). In sugar beet, a gradual increase in light intensity caused more efficient carbon flow within plants compared with sudden transitions between light and dark (Fondy et al., 1989). These differences in physiology could potentially reflect effects of altered transcriptome dynamics between plants grown under natural and laboratory conditions of light.

### Effects of Temperature Conditions in Natural Environments Upon Plant Circadian Regulation

Under natural conditions, in addition to the day/night temperature changes, there are temperature fluctuations during and between days depending on the weather conditions and season. Experiments with field-grown rice suggest that the circadian clock is robust to fluctuating environments, although the accumulation of individual transcripts is affected by temperature (Matsuzaki et al., 2015). The circadian period is relatively robust to temperature fluctuations within a specific range, typically between 12°C and 27°C, which is known as temperature compensation of the circadian clock (Gould et al., 2006). Laboratory studies have identified that PRR7, PRR9, an interaction between GI and CCA1/LHY, and FLC have roles in temperature compensation (Salomé and McClung, 2005; Edwards et al., 2006; Gould et al., 2006; Salomé et al., 2010). QTL analysis has also suggested that *ZTL* and *GI* contribute to this response (Edwards et al., 2005). Modeling suggests that blue light signaling might participate in temperature compensation through the cryptochrome photoreceptors (Gould et al., 2013). Formal quantification of temperature compensation within the circadian oscillator requires analysis of the properties of circadian rhythms under free-running conditions. However, plants under field conditions experience a greater range of varying environmental parameters in comparison to the very structured environmental conditions used to study temperature compensation in the laboratory. It is possible that responses of the circadian oscillator to the range of environmental parameters presented by field conditions provides an opportunity to investigate the environmental parameter space within which circadian rhythms remain temperature compensated.

The difference in temperature between day and night can also act as an entrainment cue. The circadian clock can be entrained by temperature fluctuations as small as 4°C (Gould et al., 2006). Daily temperature fluctuations with a warmer day and cooler night (e.g., cycles of 12 h at 31°C, 12 h at 20°C, known as thermocycles) can drive daily oscillations of 12% and 8% of genes in rice and poplar under constant light, respectively (Filichkin et al., 2011). Although many laboratory experiments investigating circadian rhythms do not incorporate day/night temperature fluctuations, such a difference can affect circadian regulation (Mizuno et al., 2014) and be essential for some aspects of plant performance. For example, a 1°C increase in the temperature during the night can decrease rice grain yield by 10% (Peng et al., 2004). Furthermore, warm night temperatures disrupt rhythmically expressed genes, particularly those genes driven by thermocyles (Desai et al., 2019), which has impacts upon productivity.

### Interactions Between Abiotic Stress Responses and Circadian Regulation Will Shape the Transcriptome Under Natural Conditions

Responses to low temperature and circadian regulation are linked intrinsically. The C-REPEAT/DRE BINDING FACTOR (CBF) proteins are major regulators of cold acclimation (Thomashow et al., 2001). *CBF1*, *2*, and *3* transcripts undergo circadian regulation in *Arabidopsis* under ambient temperature conditions, with transcript abundance peaking 4 h after subjective dawn (Harmer et al., 2000; Fowler et al., 2005). The circadian clock has two main effects on the CBF pathway; not only is there circadian regulation of basal *CBF* transcript accumulation under ambient temperatures, but there is also circadian gating of cold-induced accumulation of *CBF* transcripts. In this context, low temperature treatment causes greater *CBF* transcript accumulation 4 h after subjective dawn compared with other times of day (Fowler et al., 2005). CCA1 and LHY positively regulate the CBF-pathway because circadian cycling of *CBF* genes is absent from the *cca1 lhy* double mutants, which also have reduced freezing tolerance (Dong et al., 2011). Interestingly, a low red/far-red ratio of light causes greater CBF transcript accumulation in *Arabidopsis* in a manner that depends upon the time of day, suggesting that light quality might influence cold acclimation in a circadian-dependent manner (Franklin and Whitelam, 2007). There is also a seasonal dimension to this circadian regulation, with plants grown under short days at ambient temperatures having greater daily amplitudes of the rhythm of *CBF* transcript abundance (Lee and Thomashow, 2012). This is thought to increase freezing tolerance of plants grown under short photoperiods compared with long photoperiods (Lee and Thomashow, 2012). Furthermore, under long day conditions the CBF pathway is negatively regulated by PIF4, PIF7 and PHYB (Kidokoro et al., 2009; Lee and Thomashow, 2012). This suggests that fluctuating light and temperature, as well as photoperiod, will also impact the involvement of the circadian oscillator in the transcriptomic responses of plants to abiotic stress.

Cold acclimation responses differ between plants acclimated at constant temperatures under laboratory conditions compared with the variable temperature conditions in natural environments. For example, cold acclimation of plants in the field arises from multiple peaks of *CBF* gene expression that are concurrent with temperature decreases during the night (Hiraki et al., 2018). This contrasts the single peak of *CBF* expression during the daytime reported in plants acclimated at a constant temperature of 2°C in a growth chamber (Hiraki et al., 2018). The single or double peaks of *CBF* expression correlate with multiple Ca^2+^ signals induced by fluctuating temperatures, which is consistent with the notion that Ca^2+^ signals integrate temperature fluctuations into specific signatures that inform the expression of cold-induced genes (Kiegle et al., 2000; Hiraki et al., 2018). Photoperiod and light intensity also inform daily oscillations of cytosolic free calcium ([Ca^2+^]_cyt_) within the plant (Love et al., 2004). It is possible that under natural conditions, convergence between [Ca^2+^]_cyt_ signals induced by light, temperature, circadian regulation, and other perturbations produce specific transcriptional dynamics within the circadian oscillator that have downstream effects upon the transcriptome.

When investigating molecular aspects of plant abiotic stress responses using laboratory experiments, control experimental conditions are necessary but might lead to artefacts in the interpretation of transcriptomic responses to the environment under natural conditions. For example, in the context of low temperature responses, plants in the laboratory are not usually exposed to daily fluctuating temperatures before exposure to cold. Furthermore, cold temperature treatments are usually applied rapidly, with plants transferred from ambient to cold temperatures within a matter of minutes, whereas this is unlikely to occur under natural conditions (e.g., **Figure 2E**). Under natural conditions these previous environmental fluctuations, combined with longer term epigenetic storage of environmental information (Aikawa et al., 2010), might modify the transcriptomic response to current environments such that interpretations of transcriptional responses from laboratory conditions might not translate directly into transcriptomic responses under natural conditions.

### Contribution of Alternative Splicing to the Rhythmic Transcriptome Under Natural Conditions

An essential mechanism of transcriptome and proteome regulation in plants is alternative splicing (AS). This is the process whereby more than one transcript can be generated from a single precursor mRNA (pre-mRNA) through the use of alternative splice sites in response to developmental cues, environmental cues, and stresses (Reddy et al., 2013). These alternative transcripts can contain premature termination codons (PTCs) that cause targeting by the nonsense-mediated decay (NMD) pathway, therefore regulating the amount of coding mRNA present (Hug et al., 2016). AS can also produce proteins with altered length or domain arrangements, impacting their function or stability, so expanding the proteome diversity (Syed et al., 2012). It is thought that approximately 61% of intron-containing genes undergo AS in *Arabidopsis* (Marquez et al., 2012). AS occurs extensively across core genes of the circadian oscillator (Romanowski and Yanovsky, 2015), with mutations in AS genes leading to improper processing of clock genes. For example, a mutation in the *STIPL1* and *PRMT5* genes, which participate in the regulation of AS, alters the accumulation of circadian clock transcripts due to less efficient splicing of *PRR9* and causing a longer circadian period (Sanchez et al., 2010; Jones et al., 2012).

Recent analysis of the *Arabidopsis* transcriptome highlights a massive and rapid differential AS in response to cold temperature exposure, including a substantial proportion of circadian clock associated genes (Calixto et al., 2018). Splicing patterns of *CCA1* transcripts alter in response to biotic and abiotic cues such as pathogen exposure, cold and high light, causing the accumulation of the long intron-retaining *CCA1* isoform as well as a phase delay (Filichkin et al., 2010; Filichkin et al., 2015). Upon transfer to 4°C, the abundance of functional *Arabidopsis* LHY protein was reduced due to retention of the first intron in the *LHY* 5’-UTR, resulting in NMD (James et al., 2012a). AS of the *myb*-domain transcription factor *RVE8* as well as barley orthologs of *LHY* and *PRR7* also occurs in response to low temperature (James et al., 2012b; Calixto et al., 2016). Furthermore, a recent study showed that in sugarcane under field conditions, AS events of clock genes relates closely to temperature fluctuations, with splice forms expressed more highly at low temperatures (Dantas et al., 2019a).

There is also evidence that AS might contribute to the temperature compensation of circadian regulation (James et al., 2012a; James et al., 2012b). In *Arabidopsis*, the spliceosomal assembly factor GEMIN2 attenuates the effects of temperature on circadian period by regulating AS events. Due to differences in the splicing of clock genes in GEMIN2 mutants exposed to low temperatures for 24 h (Schlaen et al., 2015), it has been suggested that AS acts to continuously adjust the circadian clock in fluctuating temperatures under natural conditions (Dantas et al., 2019a). This means that a natural environment with fluctuating light and temperature conditions may drive alterations in AS that lead to distinct transcriptome and proteome profiles depending on the environmental conditions.

### Circadian Regulation of the Transcriptome in Crops Under Natural Conditions

An important aim of plant sciences research is to contribute to the development of the next generation of crop varieties through breeding, genetic modification, and gene editing. The incredibly pervasive influence of circadian regulation upon plant metabolism and physiology means that the circadian oscillator affects traits of agricultural importance, with a large number of oscillator components being identified as important determinants of agricultural traits (Bendix et al., 2015; Nakamichi, 2015). Translation of laboratory research concerning circadian rhythms into information of agricultural benefit requires an understanding of circadian regulation in crops under both artificial and natural environments. Understanding circadian regulation in crops provides opportunities to alter the latitudinal range of cultivation (Müller et al., 2015; Nakamichi, 2015), intercept developmental and signaling processes including photoperiodism (Turner et al., 2005; Nakamichi, 2015) and stress responses (Fowler et al., 2005; Bieniawska et al., 2008; Nakamichi et al., 2009; Seo et al., 2012; Nakamichi et al., 2016), manipulate plant defences or pollination biology (Roden and Ingle, 2009; Goodspeed et al., 2012; Yon et al., 2017a; Yon et al., 2017b), and fuse chemical biology and circadian biology to develop and optimize agrochemical use (Belbin et al., 2019; Uehara et al., 2019). On the other hand, plant breeding that causes inadvertent alterations in circadian clock function could have deleterious consequences for crop performance.

Daily programs of transcriptional regulation have been investigated in several crop species growing under natural conditions; particularly rice, sugarcane, and pineapple (Izawa et al., 2011; Nagano et al., 2012; Matsuzaki et al., 2015; Ming et al., 2015; Dantas et al., 2019a; Dantas et al., 2019b). Fewer studies have directly tested roles for circadian clock components in crops under field conditions. In one study, field-grown rice plants harboring a null mutation of *GIGANTEA* were found to have altered flowering time, stomatal conductance, fertility and grain weight compared with the background line, with the *GIGANTEA* mutation altering the expression patterns of about 75% of the transcriptome (Izawa et al., 2011). Furthermore, under field conditions this *GIGANTEA* mutant has altered accumulation of transcripts associated with GA and auxin signaling, and potential alterations in ABA and jasmonate signaling (Itoh and Izawa, 2011). When considered as a whole, the circadian oscillator of field-grown rice appears to be relatively robust to environmental fluctuations, even though individual genes have transient responses to environmental changes (Matsuzaki et al., 2015).

Tobacco is another important crop where roles for circadian clock genes have been investigated in wild relatives. Manipulations of the circadian oscillator of a wild tobacco species (*Nicotiana attenuata*) have provided insights into roles for circadian regulation under naturally fluctuating conditions, with a focus upon ecology and ecophysiology. For example, antisense-mediated misexpression of *NaLHY* and *NaTOC1* identified potential roles for the circadian oscillator gating photosynthetic responses to light under field conditions (Joo et al., 2017). The *N. attenuata* circadian oscillator also appears to contribute to the rate of flower opening and flower angle (Yon et al., 2016). Although circadian regulation modified flower selection by moths under laboratory conditions (Yon et al., 2016), these findings did not extrapolate with statistical significance to field conditions (Yon et al., 2017b). This illustrates how roles for circadian regulation can differ between field and laboratory conditions, and the need to extrapolate cautiously from the laboratory to field.

The major seed crop sunflower also gains benefits from circadian regulation. Sunflower plants track the sun such that they point eastwards around dawn and west at dusk (Vandenbrink et al., 2014). Disruption of this solar tracking in pot-grown plants in the field, by rotating pots daily so that the plant orientation was incorrect, reduced biomass and leaf area compared with correctly orientated controls (Atamian et al., 2016). In addition, correctly orientated sunflower heads received more pollinator visits than incorrectly orientated sunflowers. This is in part due to greater solar warming of the flower heads of correctly orientated plants (Atamian et al., 2016). It will be fascinating in future to identify which sunflower homologs of circadian oscillator components drive the differential growth response that underlies this mechanism (Atamian et al., 2016).

Quantitative genetics is providing valuable insights into the relationship between circadian regulation and phenotypic characteristics under laboratory and field conditions. For example, laboratory experiments have demonstrated that allelic variation in *GIGANTEA* leads to variations in the circadian period in *B. rapa* (Xie et al., 2015). In addition, experiments using a recombinant inbred line population of *B. rapa* revealed a correlation between circadian regulation, stomatal conductance, and CO_2_ assimilation, but not with reporters for water use efficiency (Edwards et al., 2012). This could suggest that circadian regulation does not contribute to drought responses associated with gas exchange traits (Edwards et al., 2012), although we note that water use efficiency under well-watered conditions is not necessarily an indicator of drought tolerance (Blum, 2009). In a separate study, the QTL that determine characteristics of circadian regulation (e.g., period and phase) were investigated in a RIL population of *Arabidopsis* (Rubin et al., 2019). Seedlings of the RIL population were entrained under field conditions at several times of year, and circadian rhythms were subsequently investigated in the laboratory using bioluminescence imaging. Intriguingly, this identified that the QTL determining the circadian phase differed between each season of entrainment (Rubin et al., 2019). While these population genetics studies did not investigate the dynamics of the entire transcriptome under field conditions, in future, it could be informative to apply circadian transcriptome analysis protocols to RIL populations under natural or laboratory conditions to better understand the mechanistic relationship between QTLs identified, changes in circadian regulation, and physiological outcomes.

A key challenge for investigations of rhythmic transcriptional regulation in crop species under natural conditions is the complexity of some crop genomes, particularly due to polyploidy. While sequencing technologies combined with analytical tools such as HomeoRoq (Akama et al., 2014) can allow quantification of transcripts derived from distinct gene homeologs in polyploids, it is also the case that the level of replication and/or sampling frequency required for timecourse studies can be high. When combined with the sequencing depth that is required to distinguish gene homeologs, sequencing cost constraints might arise.

## Perspectives

**Figure 3:**
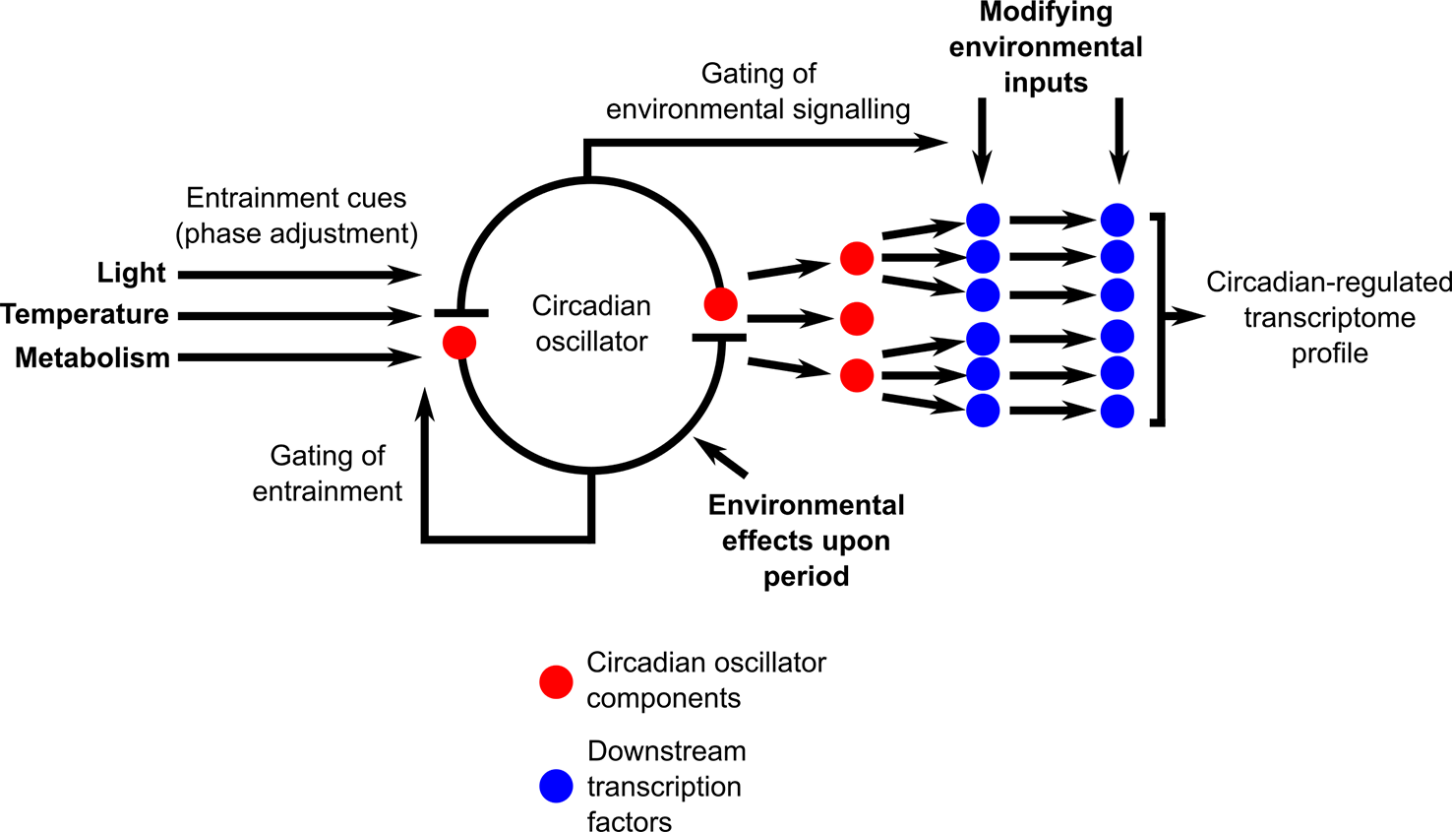
Circadian-regulated processes receive multiple environmental inputs, leading to complex interactions between circadian regulation and environmental conditions upon the transcriptome under natural conditions. Diagram conceptualizes the circadian system as a core circadian oscillator that is entrained by environmental cues and that produces output timing signals that regulate the transcriptome through a transcription factor cascade. Bold-face text indicates potential sites of environmental modification of circadian regulation, with environmental cues acting upon both oscillator inputs, oscillator function, oscillator outputs, and upon the sensitivity of environmental signaling through the process of circadian gating. In this conceptual scheme, circadian oscillator components that are transcriptional regulators (red circles) also function as the initial output step from the circadian oscillator that regulates key downstream transcription factors. In *Arabidopsis*, this role is fulfilled by oscillator components such as CCA1, LHY, the PRR, GI, and the evening complex (Nakamichi et al., 2012; Liu et al., 2013; Nagel et al., 2015; Liu et al., 2016; Ezer et al., 2017; Adams et al., 2018; Nohales et al., 2019).

Naturally fluctuating conditions impose an extraordinarily complex set of modifications to the interactions between the circadian oscillator and its outputs (**Figure 3**). There are multiple positions within the circadian system where signals communicating environmental information will modify the functioning of the circadian system (**Figure 3**). This includes the entrainment mechanisms, direct effects of the environment upon the circadian oscillator, and circadian gating of environmental signaling and response pathways (**Figure 3**). There are also direct effects of environmental cues upon circadian-regulated transcripts that are downstream of the circadian clock, such that circadian and environmental inputs are integrated separately by the transcripts (**Figure 3**). Furthermore, cascades of transcriptional regulation having a hierarchical organization will provide multiple additional positions for the entry of environmental cues into the circadian-regulated transcriptional cascades (**Figure 3**). This will lead to a continuous and dynamic adjustment of both the circadian oscillator and its outputs (Webb et al., 2019) through the action of these multiple signal inputs, with this dynamic adjustment itself altering in response to a naturally fluctuating environment. We suggest that there is future scope to extend beyond the application of transcriptional analysis under field conditions to daily cycles of integrated “omics” measures such as epigenetic states, protein abundance, and post-translational modifications and fluctuations of key primary and secondary metabolites. Furthermore, additional investigation of mutants and gene-edited plants grown outdoors in the context of circadian regulation is likely to be exceedingly informative. Advances in growth chamber technology such as fully programmable solar simulation will allow systematic investigation of the contributions of specific complex environmental parameters to circadian regulation and its outputs, thereby helping to integrate our interpretations of results from field and laboratory experiments. An interesting challenge arising from such investigations in naturally occurring plant populations is that the study species will occupy different developmental stages in distinct seasons, adding to the complexity of comparing circadian regulation between, for example, the rosette and cauline leaves of model brassica species. There is clear evidence that statistical modeling can help to unravel these complexities of daily and seasonal transcriptional programs under natural conditions (Aikawa et al., 2010; Nagano et al., 2012; Satake et al., 2013; Hepworth et al., 2018; Nagano et al., 2019). Such approaches could be invaluable to future plant sciences research as they have the potential to predict effects of future climates upon circadian function and resultant impacts upon crop and ecosystem performance.

## Author Contributions

PP and AD conceived and planned the article. PP, TM, DC-C, CG, AY, HK and AD wrote the article. TM and HK collected field environmental condition data.

## Funding

The authors thank the BBSRC (BB/J014400/1), The Leverhulme Trust (RPG-2018-216), Consejo Nacional de Ciencia y Tecnología Mexico (Conacyt), the University of Bristol, and the Bristol Centre for Agricultural Innovation for funding.

## Conflict of Interest

The authors declare that the research was conducted in the absence of any commercial or financial relationships that could be construed as a potential conflict of interest.
